# Wound Healing Potential of a Novel *Sedum* Species: *S. album Murales*

**DOI:** 10.3390/life14080958

**Published:** 2024-07-30

**Authors:** Francesca Truzzi, Elettra Frassineti, Camilla Tibaldi, Eros D’Amen, Giovanni Dinelli

**Affiliations:** Department of Agricultural and Food Sciences, University of Bologna, Viale Fanin, 44, 40127 Bologna, Italy; elettra.frassineti2@unibo.it (E.F.); camilla.tibaldi2@unibo.it (C.T.); eros.damen2@unibo.it (E.D.); giovanni.dinelli@unibo.it (G.D.)

**Keywords:** wound healing, fibroblast migration, *Sedum* species, *S. telephium*, *S. album Murales*, mucilage, polyphenols, antioxidant activity

## Abstract

Natural wound healing products are in increased demand. The potential for unexplored *Sedum* species in wound healing was discovered based on benefits of the genus reported in traditional medicine. The objectives were to screen ten *Sedum* species for wound healing, to ascertain the optimal harvest period using the five best, and finally to investigate effects of extraction protocols on wound healing using the most promising species. Different protocols were used to extract leaf polyphenol and mucilage content. Wound healing was assessed from L929 fibroblast migration. April was the optimal harvest month for wound healing efficacy, whereas the highest polyphenol content and antioxidant activity were evident in September and November. *S. album Murales* (ALBU), the best candidate, was then compared with *S. telephium* (TELE), which is well recognized in skin care. The mucilage-containing aqueous extract of ALBU was shown for the first time to induce the highest fibroblast migration after 24 h, not evident in TELE. Moreover, functional constituents contained within the absolute acetone- and isopropanol-containing polyphenol pools from ALBU induced significantly higher migration compared to TELE. A prototype cream, containing the water- and solvent-extracted bioactive compounds was effective at inducing fibroblast migration at 24 h in ALBU. The potential of ALBU in wound healing was evidenced and warrants further investigation.

## 1. Introduction

The increasing demand for natural skin care products is evident from the Natural Skin Care Products Global Report of 2024 [1] as well as numerous scientific reviews highlighting knowledge gaps and outlining future research requisites. The driving force behind the demand and interest in natural skin care products is attributable to various aspects, including increased customer interest and awareness of the multifaceted efficacy of plant products (including moisturizing, antimicrobial, anti-inflammatory, regenerative, wound healing and photoprotective properties), confirmation of the efficacy of plant constituents by scientific studies, rising skin health concerns, preferences for safer alternatives to synthetic products, preferences for ethical and environmentally sustainable source ingredients, and social media input [1,2]. Within the framework of skin care, there is also an increased demand for wound healing, with a projected 2023 to 2028 market compound growth rate (CGR) of 7.7% [3]. The increased demand in this sector is attributed to the rising global incidence of both acute (trauma- and surgery-related injuries) and chronic wounds (both lifestyle and aging population related [diabetes, pressure ulcers]) [3].

Given the demand for natural wound repair products, recent research has focused on both the screening of botanicals and the further characterization of candidate plants of interest. In recent reviews, the efficacy of current candidate plants of interest in wound care was attributed to the anti-inflammatory, antioxidant and antimicrobial properties of phytochemicals (with most attention centered on polyphenol compounds) involved in the four stages (hemostasis, inflammation, proliferation, and remodeling) of the wound healing process [4,5,6,7]. In the Mediterranean region, there is a requisite for the investigation of medicinal and aromatic plants in wound healing [5]. Interestingly, only a single reference was made to the genus *Sedum* (stonecrops) of the Family Crassulaceae [5]. For centuries, this genus has been widely used in folk medicine in many parts of the world as an anti-inflammatory, antioxidant and anti-bacterial agent in the treatment of different disease and skin ailments (including wounds, abscesses, burns and sores) [8,9]. In Italy, *S. telephium* L. is the most documented *Sedum* species in both traditional and modern herbal medicine for the treatment of wounds and burns [8,10]. 

Noteworthy, of the 350–500 *Sedum* species, only 15 have been considered even adequately investigated for phytochemical constituents and biological activity in published studies encompassing all disease-type settings [9]. This highlighted the need for more in-depth scientific-based investigations on the *Sedum* species for potential beneficial health properties especially given the widely documented reputed benefits in traditional medicine, including wound healing. Of the 15 species cited, *S. sarmentosum* Bunge (anti-aging plus hydration properties) and *S. telephium* L. (healing properties) are currently found in skin care products as well as in patented formulations advertised on the internet. *S. acre* L. and *S. telephium* L. have been specifically reported in wound healing [9]. 

A preliminary study using five spontaneous *Sedum* species in Italy, showed that the wound healing capacity was significant for all varieties investigated [8]. Given the potential for skin healing, the need for further phytochemical and biological investigations was highlighted [8]. In order to further investigate the medicinal potential of neglected *Sedum* species [9], the objective of the present study was firstly to screen 10 naturally occurring *Sedum* species for wound healing capacity by monitoring fibroblast migration towards re-epithelialization, regarded as the hallmark of wound closure [11]. The next objective was to establish whether wound healing efficacy varied according to harvest period and how wound healing compared with the expression of polyphenol content and antioxidant activity. Thereafter, the best *Sedum* species candidate was selected for more comprehensive comparisons together with the well-recognized *S. telephium* L. The present study was then aimed at assessing wound healing capacity between the two selected *Sedum* species using different extraction protocols to maximize both polyphenol and mucilage content sources. The reason was that polyphenol content in *Sedum* species was previously shown to vary significantly based on the extraction protocol [12,13] and that mucilage content, shown to possess healing properties in other succulents [14,15], has not been investigated in *Sedum* (besides *S. telephium* L.). From the polyphenol and mucilage extracts, a preliminary prototype cream for wound healing was then investigated.

## 2. Materials and Methods

### 2.1. Chemicals

Reagents for cell cultures, such as Dulbecco’s Modified Eagle Medium (DMEM), Hanks’ Balanced Salt Solution (HBSS), Fetal Bovine Serum (FBS), L-glutamine and penicillin-streptomycin and trypan blue were from GIBCO (Waltham, MA, USA). The 3-[4,5-dimethylthiazol-2-yl]-2,5 diphenyl tetrazolium bromide (MTT) was from Life Technologies (Carlsbad, CA, USA). Ethanol, acetone, isopropanol and water (HPLC-grade, Lichrosolv^®^) were purchased from Merck (Darmstadt, Germany). All other chemicals and solvents were of analytical grade.

### 2.2. Plant Material and Cultivation

The study took into consideration 10 European/Eurasian taxa of the genus Sedum, which were considered as Italian representatives. Authentication was performed by a botanical specialist G. Dinelli. Plants were authenticated from species criteria, including leaf and flower morphology, as reported in Stephenson [16]. The following 10 *Sedum* species were used: *S. acre*, *S. acre yellow*, *S. album Murales*, *S. herbstfreude*, *S. hispanicum*, *S. montanum*, *S. reflexum* or (*S. rupestre*), *S. sediforme*, *S. spectabile* and *S. telephium*. Plants were planted in 10 different plots on an experimental green roof of the Department of Agrofood Sciences and Technologies of the University of Bologna in October 2018. Mulching was performed using white stones to avoid competition with weeds, which were removed manually. Emergency irrigation was performed once per week from June to September, where a constant volume (40 mm) of water was administered. Leaf material was harvested in the mornings of the harvest months: April, May, June, September and November (2019) and frozen in liquid nitrogen. Samples were maintained at −20 °C until extraction. After the COVID period in 2022, the leaf material of the two species of interest was harvested in the Spring based on previous results. 

### 2.3. Extraction of Sedum Species in Ethanol, Water, Acetone and Isopropanol

For the selection of the best species and harvest month, leaf material was extracted in 70% (*v*/*v*) ethanol. The frozen sample (10 g) was extracted in 20 mL 70% ethanol, homogenized with a Turrax mixer and centrifuged for 10 min at 6000× *g* and the supernatant was collected to yield the extracts. For the two selected species of interest, additional extraction procedures were included. For the aqueous extracts, leaf material (20 g) was homogenized in 20 mL sterile distilled water and heated for 1 h at 100 °C. Thereafter, samples were centrifuged for 7 min at 8000× *g* and the supernatant collected. Extraction in absolute acetone and isopropanol, respectively, was performed by adding 10 g of leaf material to 10 mL to the solvents and homogenized. The homogenates were then sonicated for 10 min and filtered through sterile gauze to remove the pellet. All samples used in fibroblast, migration, proliferation and vitality assays were filtered with a 45 µm filter under sterile conditions. 

### 2.4. Preparation of the Preliminary Prototype Cream 

Xanthan gum was diluted at a concentration of 7.5 mg/mL in sterile distilled water. For the aqueous extract, the sample was added to the Xanthan gum solution at a concentration of 1:20 and stirred overnight with a magnetic stirrer at room temperature. Regarding the solvent extracts, the samples were previously mixed and evaporated and the pellet resuspended in 1 mL isopropanol. Then the resuspended samples were added to the Xanthan gum solution at a final concentration of 1:40 and stirred well for 10 min at room temperature. All extractions were performed in triplicate. Prior to use for fibroblast migration and vitality assays, the samples were filtered with a 45 µm filter under sterile conditions.

### 2.5. Measurement of Polyphenol Content, Antiradical (DPPH) Activity and Antioxidant (FRAP) Activity

The supernatants of the ethanol, water, acetone and isopropanol extracts were used for the measurement of the polyphenol content, antiradical (DPPH) and antioxidant (FRAP) activity, respectively. Polyphenols were measured according to the Folin-Ciocalteau spectrophotometric method [17]. Samples (40 μL or alternatively solvent for the blank) were added to 1.6 mL distilled water. Folin reagent (100 μL) was added for 5 min, followed by 300 μL 20% sodium carbonate (Na_2_CO_3_) solution. Samples were dark incubated for 2 h at 25 °C and then measured at 765 nm. Concentrations were calculated from a standard curve prepared with gallic acid (GAE, 0–500 mg/L) as a reference standard and the results were expressed in mg GAE (Gallic Acid Equivalents) of extract (fresh weight). The DPPH assay was performed by measuring the reduction of DPPH• to 1,1-diphenyl-2-picryl hydrazine based on the method of Floegel et al. [18]. A 0.1 mM DPPH• solution was prepared in 80% methanol. The solution was adjusted to an absorbance of 0.650 at 517 nm using 80% methanol. The extracts (50 μL sample) were then mixed with 2.95 mL of the adjusted DPPH solution, and the mixtures dark incubated at 25 °C for 30 min. Thereafter, the absorbance was measured at 517 nm against a blank containing only solvent in methanol. A calibration curve was prepared using Trolox diluted in methanol (0–500 ppm) and the final antioxidant activity was measured as %RSC (radical scavenging capacity) compared to the negative control. The FRAP method used was based on the antioxidant-mediated reduction of the ferric 2,4,6-tripyridyl-s-triazine complex (Fe^3+^ -TPTZ) to the ferrous form (Fe^2+^ -TPTZ) [19]. Samples (80 μL) or the blank (containing only solvent) were added to a 2.4 mL working solution, composed of 300 mM acetate buffer (pH 3.6), 10 mM 2, 4, 6-tripytidyl-s-triazine (TPTZ) solution and 20 mM FeCl_3_⋅6H_2_O, respectively, in a 10:1:1 ratio. The solutions were dark incubated for 1 h and measured at 593 nm. Concentrations were calculated from a standard curve prepared from FeSO_4_⋅7H_2_O (0–1000 μmol/L) and the results were expressed as μmol Fe^2+^ of extract (fresh weight).

### 2.6. Extraction and Measurement of the Mucilage Content

The extraction and measurement of the mucilage content was based on the method of Monrroy et al. [20]. Each 10 g sample was dried to a constant weight at 40 °C in a desiccator. The dried sample was ground with a motor and pestle and 1 g of the mucilaginous polysaccharide flour added to 10 mL water in pre-weighed beakers and allowed to rehydrate for 24 h at room temperature. The mucilage content was precipitated with 30 mL 100% ethanol for 30 min and the solutions centrifuged at 1000 rpm for 5 min. The supernatant was then discarded, and the pellet poured into the same previously calibrated beakers. Finally, the beakers were oven-dried at 60 °C until completely dry and the beakers weighed with the dry mucilage. The weight of the mucilage was calculated on a gravimetric basis, reflecting the difference between the weight of the beaker with the mucilage and the weight of the beaker alone. The result was expressed as a percentage (%gr) according to the formula: actual weight × 100/initial weight of the dry sample. Mucilage content extractions were performed in triplicate.

### 2.7. Fibroblast Cell Line and Growth Maintenance Conditions

L929 mouse fibroblasts (ATCC-CCL1) were cultured with DMEM, composed of 10% FBS, 1 mM L-glutamine and 1% penicillin-streptomycin. Stock cultures of the lines were maintained at 37 °C in a humidified atmosphere containing 5% CO_2_ in tissue culture flasks (75 cm^2^; BD Biosciences), and the culture medium changed every two days. Prior to use in the experiments, 20 µL cells in DMEM was added to 20 µL Blue Trypan solution as described previously [21]. Thereafter, 20 µL of the mix was removed and evaluated for cell density microscopically using a Bürker counting chamber (Blaubrand, Merck s.p.a., Milan, Italy) The correct cell densities were calculated for use in the respective wound healing assays, proliferation (MTT) and vitality (Bue Trypan) assays. 

### 2.8. Wound Healing Assay

The wound healing assay used was based on the method of Rodriguez et al. [22]. A total of 2 × 10^5^ L929 fibroblasts were plated a onto 24-well tissue culture plate and incubated until confluence for 24 h. Cells were washed three times with 0.5 mL of HBSS. Then, HBSS was aspirated from each well and a vertical cut (scratch line) was made with the tip of a 10 μL pipette to produce a slash. Plates were washed twice with HBSS to remove all detached cells and incubated with samples. For the selection of the 10 *Sedum* species, samples contained a set concentration of 10 µg/mL GAE or 70% ethanol as the CTRL. For the two selected species, 100 µL of the water, acetone, isopropanol and mixed extracts were suspended in 900 µL DMEM. Contained in each of the 100 µL volumes (for the water and solvent solutions) was 1 mg of fresh leaf material as determined from Section 2.3. For the CTRLs, the fibroblasts were not treated with extracts but contained only extraction solvent. The plates were visualized under using an inverted microscope (Eclipse Ts2, Nikon, Boston Industries, Walpole, MA, USA) at a magnification of 20× and photographed. CTRL, time zero pictures were taken. Then, the plates were placed in an incubator for a period of 4, 24 and 48 h, respectively. Fibroblast migration was assessed by one of two means. Firstly, the migration into the scratch representing the closure of the injury was assessed from measuring the distance to the scratch. Secondly fibroblast migration into the scratch was visualized under the microscope and the cells were counted manually. The results of each experiment were expressed as either as the closing distance of the scratch or the mean number of migrated cells over the set time period. The number of migrated cells of the CTRL over each specific time period was set to 100% as a basis and compared to the treatments as a percentage of the CTRL. For each sample analyzed, measurements were made from six different areas. The final results were expressed as the mean of three different experiments. 

### 2.9. Cell Proliferation (MTT) Assay

For the measurement cell proliferation study, the MTT assay was used according to the ISO 10993-5 International Standard procedure [23]. This standard protocol was based on the reduction of MTT by mitochondrial dehydrogenase of intact cells to produce purple formazan. L929 cells were plated onto 96-well tissue culture plates (10^5^ cells/well) in DMEM and treated with a 10 µg/mL GAE extract for each of the five *Sedum* species harvested in different months or with 70% ethanol as the CTRL. After incubation for 24 h, the MTT substrate (5 mg/mL phosphate buffer solution) was added to wells to attain a final concentration of 1 mg/mL. The cells were then then incubated at 37 °C in 5% CO_2_ for 2 h. After incubation, the medium was removed by aspiration. Isopropanol (100 μL) was added to each well and formazan dye formation was evaluated by a multi-well scanning spectrophotometer (Labsystems Multiskan MS Plate Reader, ThermoFisher Scientific, Waltham, MA, USA) at 540 nm. Results were expressed as the percentage of viable cells with respect to untreated controls (70% ethanol). The percentage of cell proliferation was calculated using the following formula: absorbance value of treated sample/absorbance value of control × 100 = % of cell viability.

### 2.10. Cell Viability

Vitality was measured using the blue trypan assay [24]. L929 cells were plated onto 24-well tissue culture plate (5 × 10^4^ cells/well) in complete medium for 24 h until confluency. Thereafter, the water, acetone and isopropanol extracts from ALBU and TELE were diluted in DMEM at different concentrations (1:30, 1:40, 1:50, 1:100) for 24 h. To detect viability, cells were then carefully resuspended in a 0.4% Trypan Blue (Gibco) solution and vital cells were counted using Countess^®^II FL (ThermoFisher Scientific, Waltham, MA, USA). The results were expressed as a viability percentage of the control.

### 2.11. Statistical Analysis

Data were presented as the means ± standard deviation (SD) of experimental measurements made in triplicate. Statistical analysis was conducted using GraphPad Prism Version 9.3.1 (2021). Significance was determined by either one-way variance (ANOVA) and the Turkey-Kramer test at the 95% confidence level. For repeated measures, two-way ANOVA followed by Bonferroni’s post hoc test was used. Statistical differences were considered to be significant at *p* < 0.05.

## 3. Results and Discussion

### 3.1. Selection of the Best Sedum Species for Wound Healing

Given the potential skin healing ability of the *Sedum* species from the preliminary study reported previously, the first objective was to screen 10 species in the Emilia Romagna region of central Italy. Aside from the potential wound healing ability, extensive green roof cultivation (cost effective, light weight and low maintenance) of *Sedum* species in the Mediterranean environment has also garnered increased interest as a sustainable means to integrate vegetation in urban environments [25]. In the present study, the following 10 *Sedum* species (with abbreviations), under extensive green roof cultivation in Bologna (Italy), were examined for wound healing properties: *S. acre* (ACRE), *S. acre yellow* (ACREY), *S. album Murales* (ALBU), *S. herbstfreude* (HERB), *S. hispanicum* (HISP), *S. montanum* (MONT), *S. reflexum* or (*S. rupestre*) (REFL), *S. sediforme*, (SEDI) *S. spectabile* (SPECT) and *S. telephium* (TELE). 

Given that in the *Sedum* species, interest in the healing properties has been primarily centered on the polyphenol content (including phenolic acids and flavonoids) and associated antioxidant activity [8,9,12,13,26], leaf material from the April and May harvests were pooled and extracted in 70% ethanol for polyphenol content. However, the motivation in the present study for screening the 10 species for wound healing capacity rather than polyphenol content was based on the results of Chiocchio et al. [8]. The latter showed that the “functional” capacity of the extracts in stimulating keratinocyte migration, to measure the efficacy of the proliferation phase in wound healing, was not correlated to the polyphenol content present in the respective extracts [8]. Those authors did show that the optimal dose concentration for fibroblast migration was 10 µg/mL of gallic acid equivalents (GAE) [8]. Hence, the polyphenol content was firstly extracted and quantified in each of the 10 species. Then, the correct volumes were used to ensure a final concentration of 10 µg/mL of gallic acid equivalents (GAE) diluted in DMEM. The objective was to see from a constant concentration, which species contained functional constituents capable of stimulating fibroblast migration. This was performed by assessing the migration of L929 mouse fibroblast cells in the scratch wound healing assay. L929 cells were selected for use in this study as the cell line is recommended by ISO 10993-5 for toxicity studies and has been used by the majority of laboratories [27]. Additionally, a significant number of in vitro studies evaluating the wound healing potential of natural products were shown to use L929 mouse fibroblasts [7].

**Figure 1:**
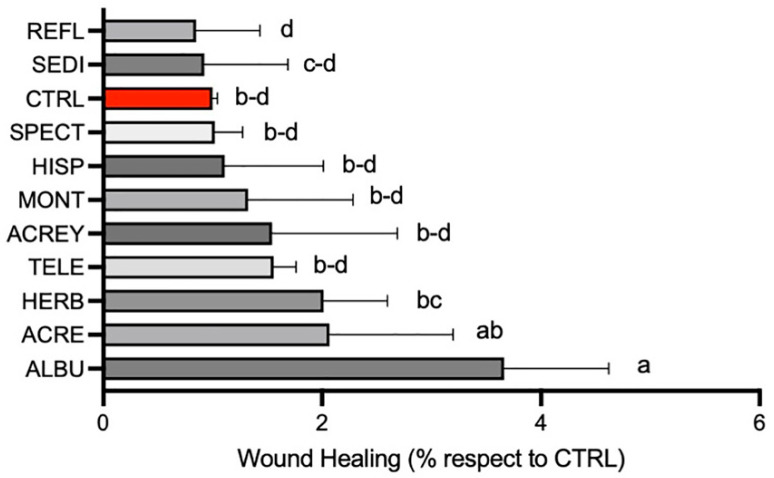
Percentage wound closure by L929 fibroblast migration following 4 h exposure to 10 µg/mL gallic acid equivalents (GAE) from 10 *Sedum* species, respectively, compared to the untreated control (CTRL). Statistical analysis was performed with the letters (a, b, c, d) representing significant differences between treatments as determined by one-way ANOVA and the Turkey-Kramer test at the 95% confidence level (*p* < 0.05). Abbreviations for *Sedum* species: *S. acre* (ACRE), *S. acre* yellow (ACREY), *S. album Murales* (ALBU), *S. herbstfreude* (HERB), *S. hispanicum* (HISP), *S. montanum* (MONT), *S. reflexum* (REFL), *S. sediforme*, (SEDI) *S. spectabile* (SPECT) and *S. telephium* (TELE).

The percentage of wound closure based on fibroblast cell monolayer migration on a 2-dimensional (2D) surface was calculated after 4 h and compared to the untreated control (CTRL) containing no *Sedum* extracts. The species was significant (*p* < 0.05) for rapid wound healing capacity (Figure 1). The extract of ALBU induced the most rapid migration of fibroblasts (4 h), not significantly different from that of ACRE, but significantly higher than those of the remaining species. Only ALBU induced a rapid wound healing capacity that was significantly higher than the CTRL (Figure 1). Results evidenced a differing functional capacity within the 10 µg/mL GAE polyphenol content for each species, evidently attributable to different individual polyphenol constituents. The involvement of other components extracted in the 70% ethanol could not be excluded. Selection of five of the ten species was purely based on choosing those species with the highest mean wound healing capacity. In descending order, the five best performing *Sedum* species were ALBU, ACRE, HERB, TELE and ACREY, respectively (Figure 1).

### 3.2. Selection of the Best Harvest Period for Wound Healing

The next objective was to further examine the impact of harvesting dates on the wound healing capacity, since changes in bioactive components by biotic and abiotic factors were highlighted as important factors requiring consideration towards maximizing bioactivity of skin formulations [7]. To this end, the effect of species, harvest month and the interaction species × harvest month on both fibroblast migration and fibroblast proliferation in the five selected species were investigated. Since polyphenol constituents, responsible for functional activity, are products of secondary metabolism and vary based on biotic and abiotic factors, it was considered important to investigate the effect of harvest month for wound healing capacity [7]. For each *Sedum* species, the mean wound healing percentages induced by leaf material extracts were extended to also include the July, September and November harvests. Since the period between December and March coincides with the well-documented reduced growth period in *Sedum* over winter, with some species displaying dormancy, plants were not harvested in this period.

**Figure 2:**
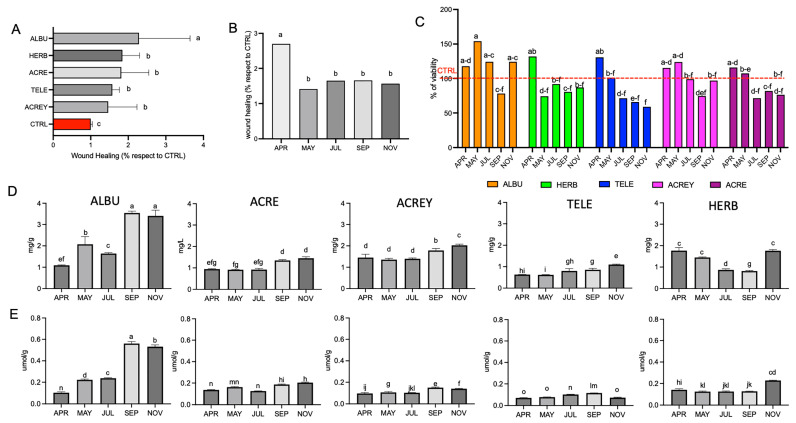
(**A**) Percentage wound closure by fibroblast migration following a 4 h exposure to 10 µg/mL GAE, representing the average of harvests conducted over five months for five *Sedum* species, compared to the CTRL and (**B**) percentage wound closure by fibroblast migration following a 4 h exposure to 10 µg/mL GAE, representing the average of five *Sedum* species, for each harvest month. (**C**) Fibroblast viability (MTT assay), expressed as percentage of the untreated CTRL (100%) for five *Sedum* species and five harvest months. (**D**) Polyphenol content (expressed in mg GAE/g) in relation to harvest month for each of the five *Sedum* species and (**E**) FRAP activity (expressed in μmol Fe^2+^/g) in relation to harvest month for each of the five *Sedum* species. (**A**,**B**,**D**,**E**) Statistical analysis was performed with the letters (a, b, c, d … o) representing significant differences between treatments as determined by one-way ANOVA and the Turkey-Kramer test at the 95% confidence level (*p* < 0.05). (**C**) Statistical analysis was performed using a two-way ANOVA at the 95% confidence level (*p* < 0.05). Abbreviations for *Sedum* species: *S. acre* (ACRE), *S. acre* yellow (ACREY), *S. album Murales* (ALBU), *S. herbstfreude* (HERB) and *S. telephium* (TELE).

A two-way ANOVA (*p* < 0.05) showed a non-significant interaction between species and harvest month on fibroblast migration. However, species alone (*p* < 0.05) was significant for wound healing (Figure 2A). ALBU extracts were shown to induce the most rapid migration of fibroblasts after 4 h with a wound healing percentage that was significantly higher than the remaining four species, with no significant differences between the latter. All species induced a wound healing capacity that was significantly higher than the CTRL (Figure 2A). Harvest month was also significant (*p* < 0.05), with April shown to be the best month to harvest leaf material for improved functional rapid wound healing capacity in all five *Sedum* species (Figure 2B). This underscores the importance of investigating changes in functional capacity, in order to maximize bioactivity of skin formulations.

Aside from fibroblast migration, examining cell viability is considered essential to ascertain whether the extracts stimulate proliferation or whether cytotoxic side effects are present. Hence, the MTT assay was performed to measure fibroblast proliferation following a 24 h exposure to extracts of the same five *Sedum* species over each of the five harvesting periods, respectively. A two-way ANOVA showed a significant interaction between species and harvest month on fibroblast migration (*p* < 0.05). For all five species, extracts harvested in April induced the highest levels of proliferation on average, compared to the remaining months, particularly September with the lowest proliferation percentages (Figure 2C). May-harvested extracts of ACREY and ACRE, and in particular ALBU, also stimulated fibroblast proliferation. Previous work has shown that selected polyphenol constituents from other medicinal plants of interest in skin healing were responsible for stimulating not only migration but also fibroblast proliferation, also considered an important aspect of wound healing [28,29,30]. Of additional relevance, extracts were not shown to be cytotoxic to the fibroblast viability when compared to the CTRL (representing 100%; *p* < 0.05).

Since the selection of candidate plants for functional studies is generally based on total polyphenol content, the latter and associated antioxidant potential using the ferric reducing antioxidant power (FRAP) assay were investigated for harvest month in the five *Sedum* species. Despite the significantly higher wound healing capacity in the month of April for all species (Figure 2B), the total polyphenol content was the highest in September and November for ALBU and ACRE, ACREY in July, November for TELE, and in April and November for HERB, respectively (Figure 2D). Abiotic stressors are widely reported to be inducers of polyphenol expression. In the present investigation (green roof cultivation), water was supplied and was not considered as a stress factor modulating the differences in polyphenol expression. Temperature and phenological stage were likely contributory factors. However, it is noteworthy that improved wound healing efficacy, was noted in the phenological period of active growth (April) in which the minimum and maximum temperatures were 6.4 and 18.6 °C, respectively. Instead, the overall higher polyphenol content and antioxidant activity (September and November) were associated with the end of flowering and pre-dormancy phenological stages of September and November, respectively. The minimum and maximum temperatures were 14.6 and 26.6 °C for September and 7.4 and 13.9 °C for November, respectively. Given that there was no correlation between wound healing capacity and total polyphenol content (compare Figure 2A,D), suggests the involvement of specific polyphenol components expressed in April or the expression of alternative components such as alkaloids. This is warranting of further investigation in order to maximize the harvest potential for functional components towards developing creams. Although FRAP activity (similar to polyphenol content) was significantly higher in September and November than April for ALBU, ACRE, and ACREY, increased FRAP activity was not associated with increased polyphenol content in TELE (Figure 2E). The results clearly evidenced that total polyphenol content per se was not a suitable selection criterion towards guaranteeing functional efficacy in stimulating fibroblast migration. This highlights the appeal made towards identifying the main compounds responsible for wound healing [7]. As opposed to total polyphenol content (Figure 2D), the effect of specific polyphenolic constituents on wound healing, predominantly expressed in April (Figure 2B) and in ALBU (Figure 1 and Figure 2A) on wound healing can be inferred.

### 3.3. Bioactive Contents in Water, Acetone and Isopropanol and Effects on Wound Healing in S. album and S. telephium

ALBU was shown to induce the highest fibroblast migration compared to the remaining species and was thus selected as the candidate *Sedum* species for further investigations. To date, ALBU remains an unrecognized *Sedum* species for biological activity of phytochemical constituents [9]. Given that TELE is recognized for wound healing properties [9], and is a medicinal species of interest in Italy [10], and is also included in a skin care patent (https://patents.google.com/patent/WO2016188919A1/zh, accessed on 29 January 2024), TELE was selected as a comparative control. 

Regarding the potential role of additional functional compound classes, *Sedum* leaves were reported to be predominantly composed of polysaccharides and polyphenol flavonols [31]. Of interest were the mucilaginous polysaccharides, large polar molecules, predominantly composed of carbohydrates and uronic acids, as well as glycoproteins and other bioactive compounds [32]. Mucilage content is gaining interest for its medicinal properties, including that of wound healing [14,15]. Interestingly, in TELE, it was previously shown that unlike the lyophilized flavonoid fraction (and total lyophilized juice), which possessed strong antioxidant/free radical scavenging properties and in vivo photoprotective effects against UVB-induced skin damage, the lyophilized polysaccharidic fraction was completely ineffective [26]. Moreover, in TELE, it was also shown that, both a more and less soluble polysaccharide fraction (not the flavonoid fraction) inhibited cell adhesion in fibroblasts (important in wound healing), suggesting that TELE polysaccharides, similar to heparin, were able to interfere with cell-matrix interactions [31]. The increased wound healing capacity of ALBU, extracted in 70% ethanol in the present study, was not attributable to mucilage content as these polymers are insoluble in ethanol, and to the best of our knowledge mucilage content and its potential skin care ability, has never been investigated.

To determine the overall functional capacity (including mucilage content) in stimulating wound healing, the objective was to ensure the extraction of all potential functional components from both TELE and ALBU. To this end, three extraction procedures were performed using water, absolute acetone and isopropanol, respectively. Water (relative permittivity of 80 ε) was selected for the extraction of mucilaginous polysaccharides and polar polyphenols. Absolute acetone, as an intermediate (moderate) polar solvent (relative permittivity of 21.5 ε), was previously shown to be significantly more effective at extracting polyphenols in *S. acre* compared to less polar alcohols (methanol) [12]. Isopropanol (relative permittivity of 18 ε) was selected for the extraction of lipid-based bioactive molecules but was also previously used in an isopropanol salting-out pretreatment to isolate specific polyphenols with high antioxidant activity from 60% methanol extracts of *S. formosanum* N.E.Br. [33]. Hence, the solvent extraction procedures selected met the requisite criteria to investigate functional performance: (1) low toxicity for administration to cells, (2) miscible with water as the extracts would be diluted in DMEM, and (3) inclusion of hydrophilic and lipophilic components. 

**Figure 3:**
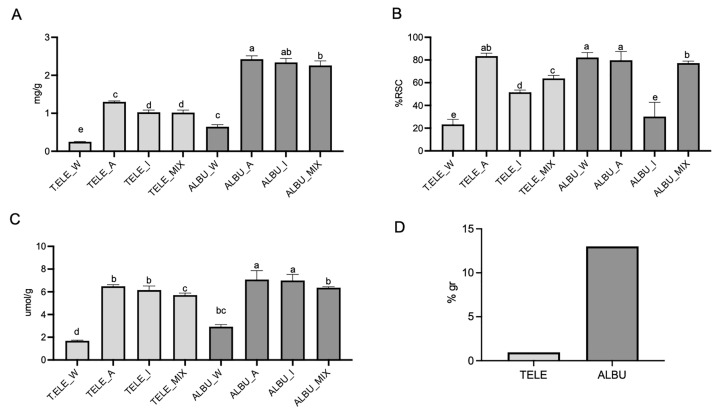
(**A**) Polyphenol content (expressed in mg GAE/g), (**B**) DPPH antiradical activity (expressed in %RSC), (**C**) FRAP activity (expressed in μmol Fe^2+^/g) and (**D**) mucilage content in TELE and ALBU (expressed in %gr). Extracts were performed in water (W), acetone (A), isopropanol (I), with mixed (MIX) extracts containing the three extraction volumes in an equivalent ratio (1:1:1). Statistical analysis was performed with the letters (a, b, c, d, e) representing significant differences between treatments as determined by one-way ANOVA and the Turkey-Kramer test at the 95% confidence level (*p* < 0.05). Abbreviations for *Sedum* species: *S. album Murales* (ALBU), and *S. telephium* (TELE).

The constituents of interest, namely the polyphenols and mucilage polymers, were then examined for both species. Firstly, the polyphenols were measured in the three different extracts as well as in a mix, comprised of an equal proportion 1: 1: 1 of water: acetone: isopropanol. As was also evident in Figure 2D, ALBU contained a significantly higher proportion of total polyphenols in all extracts compared to TELE (Figure 3A). In both species, there was also a significantly higher proportion of intermediate polar polyphenols compared to polar polyphenols (Figure 3A). Of interest, the mucilage content (only extractable in water) was significantly higher in ALBU compared to TELE (Figure 3D). The 2,2-diphenyl-1-picrylhydrazyl (DPPH) assay, measuring antiradical power, reflected the radical scavenging contribution of the mucilage content in the aqueous extracts of ALBU, not evident for TELE (Figure 3B). The lower DPPH activity in the polysaccharide fraction of TELE, and the consequent ineffective in vivo photoprotective effects against UVB-induced skin damage reported by Bonina et al. [26], may have been a reflection of the low mucilage content. 

In both species, the acetone extract contained a higher antiradical power than the isopropanol extract, even though total polyphenol content was comparable (Figure 3B). This result corroborated previous work on *S. acre* showing that acetone extracts contained higher antiradical activity than ethyl acetate, which is similar to isopropanol extracted oil-based molecules in addition to more polar polyphenols [12]. Of interest, the antiradical activity (Figure 3B) in the acetone extract of TELE was equivalent to that of ALBU, notwithstanding the lower total polyphenol content in TELE (Figure 3A). Likewise, the antioxidant activity (FRAP) in the solvent extractions between TELE and ALBU were equivalent, despite the lower polyphenol content in TELE (Figure 3C). FRAP activity in the aqueous extracts of ALBU was significantly higher than that of TELE, likely reflecting the contribution of the mucilage content. As expected, the mixes reflected the average polyphenol content and antiradical/antioxidant activity of that measured in the three different extracts depicted in Figure 3.

**Figure 4:**
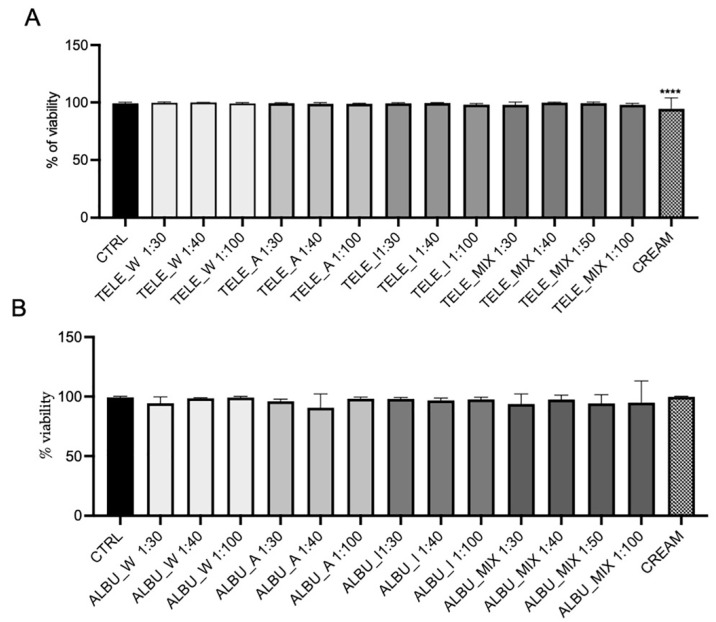
(**A**) TELE- and (**B**) ALBU-induced fibroblast vitality (Blue Trypan assay) compared to the untreated control (CTRL). Extracts were performed in water (W), acetone (A), isopropanol (I), with mixed (MIX) extracts containing the three extraction volumes in an equivalent ratio (1:1:1). All extracts were prepared at different extract: DMEM dilutions (1:30, 1:40, 1:50, 1:100), and the cream at a 1:100 dilution. Extracts were exposed to the cells for 24 h. Statistical analysis was performed with the number of stars **** representing significant differences between treatments as determined by one-way ANOVA at the 95% confidence level (*p* < 0.05). Abbreviations for *Sedum* species: *S. album Murales* (ALBU), and *S. telephium* (TELE).

The administration of extracts to the fibroblasts was standardized to an equivalent volume basis in order to account for all functional constituents contained in a set volume for both species. Firstly, the effect of the respective extracts was examined on fibroblast vitality. Given that all solvents were miscible in DMEM, a series of dilutions of extract: DMEM were made ranging from 1:30, 1:40 (1:50) to 1:100, respectively. In a 1:100 acetone: DMEM dilution, there would be approximately 6 and 13 µg/mL GAE for TELE and ALBU, respectively, which was consistent with the 10 µg/mL GAE selected as the optimal content previously [8]. Compared to the untreated CTRL, there were no cytotoxic effects on fibroblast cell vitality for the individual extracts (water, acetone and isopropanol) and mixes, respectively, derived from either TELE (Figure 4A) or ALBU (Figure 4B). The cream extracts, also depicted in Figure 4A,B will be discussed under the next subheading.

**Figure 5:**
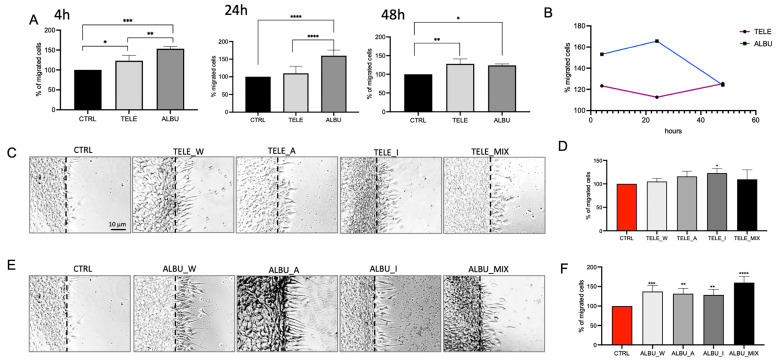
(**A**,**B**) Fibroblast migration percentages induced by mixed (MIX) extracts from ALBU and TELE after 4, 24 and 48 h compared to the control (CTRL). (**C**,**E**) Microscope images (×20 magnification) portraying fibroblast migration induced by extracts of TELE and ALBU, extracted in water (W), acetone (A), isopropanol (I), with MIX extracts containing the three extraction volumes in an equivalent ratio (1:1:1) after 24 h. (**D**,**F**) Quantification of fibroblast migration derived from the images in (**C**,**E**). All experiments were prepared at an extract: DMEM dilution of 1:100. Statistical analysis was performed with the number of stars, *, **, ***, **** representing significant differences between treatments as determined by one-way ANOVA at the 95% confidence level (*p* < 0.05). Abbreviations for *Sedum* species: *S. album Murales* (ALBU), and *S. telephium* (TELE).

ALBU had been selected as the candidate species for investigation based on wound healing capacity, but only after 4 h and only in a 70% ethanol extract (Figure 1 and Figure 2A). Using the mix of all potential functional constituents from the water, acetone and isopropanol extracts (1:1:1), the percentage fibroblast migration was examined after 4, 24 and 48 h, respectively, in both species. ALBU extracts demonstrated significantly higher fibroblast migration after 4 and 24 h compared to the CTRL and TELE (Figure 5A). Instead after 48 h, fibroblast migration for TELE increased significantly compared to the CTRL and was equivalent to that of ALBU (Figure 5A). Differences in wound healing capacity in relation to time were demonstrated previously, with wound healing increasing from 6 to 24 h with *S. acre*, *S. album* and *S. rupestre* extracts and tapering off when using *S. hispanicum* and *S. sexangulare* [8]. Performance after 48 h was not investigated in the latter species [8]. Interestingly, rapid wound healing response capacity was evident for ALBU, whereas TELE extracts showed a delayed increase manifested after 24 h (Figure 5B). 

To investigate the specific effects of the different extracts, and mixes on fibroblast migration, a single time period of 24 h was selected. For ALBU, all extracts contributed to the significantly increased migration of fibroblasts compared to the CTRL (Figure 5E,F). Noteworthy, the aqueous extract produced a more significant effect on fibroblast migration than the solvent extracts, with the mix producing a synergistically higher effect than the individual extracts. In TELE, only the isopropanol extract induced significant migration compared to the CTRL (Figure 5C,D). However, after 48 h, the increased migration (Figure 5B), after exposure to TELE, was attributable to a comparable effect of both the acetone and the isopropanol extracts.

It can be concluded that in ALBU, unlike TELE, the mucilage content with a significant anti-radical activity contributed significantly to increased fibroblast migration. This result corroborated previous work on additional mucilage-containing medicinal plants, showing that mucilage stimulates fibroblast migration in wound healing [14,15]. In addition, both the acetone and isopropanol extracts contained functional constituents contributing to fibroblast migration after 24 h for ALBU and after 48 h for TELE, respectively. The solvent extractions of ALBU induced a greater fibroblast migration after 24 h than that of TELE (Figure 5D,F), similar to that produced after 4 h in ethanol (Figure 1 and Figure 2A). Given that the isopropanol extract (also containing lipid-based molecules) did not produce a significantly improved fibroblast migration over the acetone extract suggests that specific polyphenol constituents extracted in both solvents (having a similar polarity) contributed to the functional efficacy. 

It is also possible that alkaloids, not measured in the present investigation, that would have been extracted in both the water and solvents, may have contributed to the wound healing properties. 

### 3.4. Bioactive Potential for Fibroblast Migration in a Preliminary Protype Cream Produced from the Combination of Water, Acetone and Isopropanol Extracts from S. album and S. telephium

A preliminary prototype cream was produced containing 1:20 dilution of the aqueous extract and a 1:40 dilution of the solvent-based extracts in water-containing xanthan gum from both species. To test the efficacy of the cream on fibroblast migration, a 1:100 dilution in DMEM was made and exposed to fibroblasts for 24 h to firstly investigate effects on vitality. No cytotoxic effect on cell vitality was evident in the cream prepared from ALBU extracts (Figure 4B). In contrast, a mild but significant cytotoxic effect was shown for the cream prepared from TELE extracts (Figure 4A). Thereafter, the effect of the creams on fibroblast migration was determined.

The final polyphenol content of the cream was calculated based on the contents present in both the aqueous and solvent extracts, respectively, for both ALBU and TELE in Figure 3A, and by taking into consideration the procedure for developing the cream. Similarly, the mucilage content was calculated from the aqueous extracts for the two species in Figure 3D, taking into consideration the cream preparation method. As such, in the final cream preparation for ALBU, the polyphenol and mucilage contents were 645 µg/100 mL and 325 mg/100 mL, respectively, whereas for TELE, the polyphenol and mucilage contents were 275 µg/100 mL and 25 mg/100 mL, respectively. The cream mix instead was comprised of the average polyphenol and mucilage contents of the respective individual species.

**Figure 6:**
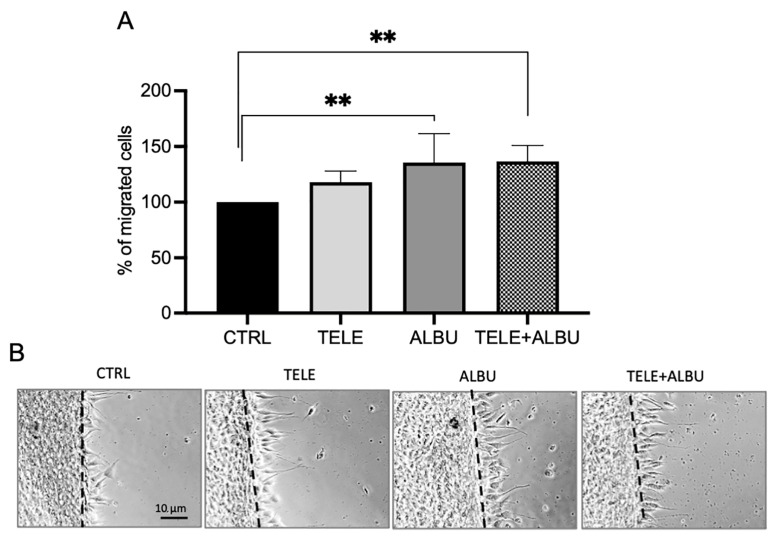
(**A**) Statistical quantification of fibroblast migration percentages induced by mixed (MIX) extracts from ALBU and TELE after 24 h compared to the control (CTRL). (**B**) Microscope images (x 20 magnification) portraying fibroblast migration induced by the MIX, containing constituents extracted in water (W), acetone (A), isopropanol (I) in a 1:1:1 ratio and then exposed to the cells at an extract: DMEM dilution of 1:100. Statistical analysis was performed with the number of stars, ** representing significant differences between treatments as determined by one-way ANOVA at the 95% confidence level (*p* < 0.05). Abbreviations for *Sedum* species: *S. album Murales* (ALBU), and *S. telephium* (TELE).

The cream produced from ALBU was effective at significantly stimulating fibroblast migration compared to the control and TELE (Figure 6A,B). This result was anticipated given the contribution of the aqueous mucilage-containing extract (absent in TELE) and the polyphenol containing acetone and isopropanol extracts (as well as ethanol) with specific functional constituents able to induce fibroblast migration (Figure 5E). Evidence of the functionality of the prototype cream is evident as the functional constituents contained within the formulation were able to induce fibroblast migration. However, given that TELE is a well-recognized species, and was shown to contain comparable antiradical/antioxidant activities to ALBU, despite a significantly lower total polyphenol content, a cream was similarly prepared containing both species in equal proportions in a 1:20 dilution. The combined species cream was shown to produce a comparable effect to ALBU alone (Figure 6A,B). Although the induction of fibroblast migration can be primarily attributed to ALBU, the inclusion of comparable contents of antioxidant/antiradical bioactive components (polyphenols) is important for the inflammation phase of wound healing associated with an increased ROS production [7]. Specific evidence for the antioxidant efficacy of TELE was shown from in vivo studies showing protection again UVB-induced skin damage [26].

### 3.5. Considerations and Future Research Requirements on S. album Murales for Wound Healing Applications

ALBU was shown to be distinctive in the contribution of the aqueous-containing mucilage component in stimulating fibroblast migration. The minimal contribution of the polysaccharides (mucilage) to wound healing in TELE, reported previously [26], was corroborated by the present study as well as a recent preprint [34]. Given the novel wound healing potential of the mucilage fraction from ALBU and its potential use in topical creams, confirmation of the efficacy of plant constituents by scientific studies is necessary [1,2]. To this end, attention must be given to the following: what components within this fraction are responsible for wound healing that can facilitate refining the extraction, when would be the optimal period to harvest mucilage components, and how would they perform on in vitro three-dimensional (3D) skin equivalents? 

To address the first question, a comparative characterization of the aqueous-mucilage fraction of both ALBU and TELE is warranted to identity differences in the composition to facilitate the identification of novel components existing in ALBU. Of potential interest are the contribution of alkaloids, which have been characterized in TELE [35] but are yet to be identified in ALBU. It is also possible that alkaloids, that would have been extracted not only in water, but also in both solvents, may also have contributed to the increased wound healing properties in the polyphenol-containing solvent fractions of ALBU. A limitation of the study was the variation in the overall mucilage content, necessitating improvements and a refinement of the aqueous extraction procedure. Approaches such as computational analysis utilizing novel approach methodologies (NAMs) to investigate the pharmacokinetic of phytochemicals was proposed by Rai et al. [36] and can also be investigated in *Sedum* species.

Regarding the optimal period to harvest mucilage, it was shown that there was an increased staining of mucilage cells in the leaves and stems of *S. aizoon* L. plants exposed to cold temperature [37]. Mucilage production was proposed to be a mechanism of low temperature tolerance [37]. Investigating not only the production of mucilage but wound healing efficacy of this component throughout the year in relation to temperature is a necessity. Though it has been recommended that plants cultivated for medicinal purposes be maintained under constant standardized growth conditions [7], cold-stress inducers of important compounds warrant an investigation to promote the feasibility of extensive green roof cultivation of *Sedum* species. This approach offers a unique opportunity to integrate sustainable cultivation with medicinal research. *Sedum* species are particularly suitable to low-input cultivation based on their shallow root systems, wide-range temperature adaptability, Crassulacean acid metabolism (CAM) type photosynthesis, drought resistance and disease tolerance [25]. A more detailed investigation of polyphenol expression compared to wound healing efficacy in relation to both temperature and phenological stages of plant development is also warranting. It is possible that harvest periods for best wound healing efficacy in the mucilage and polyphenol components will vary.

The use of physiologically more relevant 3D skin equivalents to test both basic constituents and creams is an essential preclinical requirement towards the developing creams for the natural skin repair market. The 3D skin model includes basic epidermal (keratinocytes) and dermal structures (containing fibroblasts) that can be constructed from primary cells (patient biopsies) or commercial cell-lines. Recently, the efficacy of total juice and polysaccharide components of TELE were analyzed and tested in 2D on human keratinocytes (HaCaT) and fibroblasts (HFF-1) to assess cell viability, wound closure, and the production of growth factors and pro-collagen I [34]. Whilst the mucilage component had no effect, the total juice component (polyphenols and other bio-actives) significantly enhanced wound closure in both cell types, with a marked increase in fibroblast growth factor [34].

ALBU was also shown to be distinctive in promoting fibroblast migration more rapidly (4–24 h) on the part of the absolute acetone- and isopropanol-containing polyphenol pools. In contrast, TELE showed the reverse trend, with efficacy increasing between 24 and 48 h. Differences in the time required by the absolute acetone- and isopropanol-containing polyphenol pools to induce the migration of fibroblasts between ALBU and TELE are likely to be attributable to specific polyphenol components or alternative compounds such as alkaloids contained within these pools. The complementary actions hold interesting implications for the development of a cream containing both ALBU and TELE. Although the present study shows a greater wound healing efficacy of ALBU compared to TELE, the leaf juice of TELE has the capacity for wound closure [9,33] and its well reputed anti-inflammatory, antioxidant and hydration properties have contributed to the inclusion of TELE in skin care products as well as in patented formulations advertised on the internet. Hence, the development of a topical cream containing ALBU alone or in combination with TELE is warranting future investigations as outlined above.

## 4. Conclusions

ALBU, which remains an unexplored *Sedum* species, showed the greatest wound healing potential (assessed from fibroblast migration) out of the original 10 *Sedum* species that were screened. ALBU was compared to TELE which is well recognized for its skin repair properties and is contained in skin care products. ALBU contained functional constituents, extracted in 70% ethanol, acetone and isopropanol that specifically induced fibroblast migration to a greater extent than TELE after 4 h and 24 h. Moreover, the considerable mucilage content of ALBU, not evident in TELE, induced the highest fibroblast migration. To the best of our knowledge this is the first time that an aqueous mucilage-containing extract has shown to stimulate fibroblast migration in *Sedum* species. In a preliminary prototype cream, ALBU was shown to induce fibroblast migration to a greater extent than TELE. It is feasible to conclude that ALBU has potential for use in skin-repair creams. However, more research is needed in order to meet market requirements. Such research includes the characterization of the mucilage fraction which can be compared to TELE and is lacking in the components to induce wound healing. Moreover, it is essential to investigate the optimal harvest period (in relation to abiotic factors and plant developmental stages) specifically for wound healing efficacy in the mucilage and polyphenol fractions of ALBU. Investigating the wound healing efficacy of the polyphenol and mucilage components in prototype creams on 3D in vitro physiologically relevant skin models is also a necessity. 

## Data Availability

Data are unavailable due to privacy.
